# Perceptions of South Africa’s master of public health graduates on the degree’s contribution to their leadership at work and in society

**DOI:** 10.3389/fpubh.2025.1620477

**Published:** 2025-10-08

**Authors:** Virginia Zweigenthal, Nicola Christofides, Thembelihle Dlungwane, Sogo France Matlala, Mathildah Mpata Mokgatle, Abraham Opare, Sean Mark Patrick, Nikki Schaay, Maylene Shung-King, Takalani Tshitangano, Laetitia Rispel

**Affiliations:** ^1^School of Public Health, University of Cape Town, Cape Town, South Africa; ^2^School of Public Health, University of the Witwatersrand, Johannesburg, South Africa; ^3^Discipline of Public Health Medicine, University of KwaZulu-Natal, Durban, South Africa; ^4^Department of Public Health, Sefako Makgatho Health Sciences University, Pretoria, South Africa; ^5^School of Health Systems and Public Health, University of Pretoria, Pretoria, South Africa; ^6^School of Public Health, University of the Western Cape, Cape Town, South Africa; ^7^South African Research Chairs Initiative (SARChI), Johannesburg, South Africa

**Keywords:** public health workforce capacity building, public health workforce training, South Africa, public health leadership, low-and middle-income countries

## Abstract

**Introduction:**

Integrating public health functions into national health systems is essential to enhance population health. The Master of Public Health (MPH) degree is an important foundation for public health practice in low-and middle-income countries such as South Africa. However, insufficient evidence on individual motivations for undertaking the MPH and the perceptions of graduates on the utility of the degree at work and in society and its contribution to their leadership skills informed this study.

**Methods:**

A consortium of academics from eight South African universities developed a self-administered questionnaire to measure inter alia the socio-demographic characteristics, motivations, career paths, perceptions of the utility of the degree, and its contribution to their professional and personal development. The study population comprised the 2012–2016 cohort of MPH graduates from eight universities. Following informed consent, eligible graduates completed an online survey via REDCap. The data were analyzed using Stata.

**Results:**

A total of 221 graduates completed the survey. The mean age of respondents was 35 years, and the majority were from South Africa (53.2%) or other African countries (43.2%). The majority (91.1%) completed the MPH to improve their skills or to promote their personal development for senior management and leadership roles. Approximately 75% used identified leadership skills at work, but only half these skills were obtained from the MPH. Over 80% of respondents positively impacted on their workplace and in society, using skills mostly derived from the MPH in all domains.

**Discussion:**

This cohort of MPH graduates exercised leadership in different settings, but many stated that these skills were not obtained from the MPH programs. The COVID-19 pandemic underscored the need for public health leaders skilled in communication, collaboration, and crisis management, amidst considerations of social justice and equity. Hence, leadership skills need to be intentionally included in MPH programs in South Africa.

## Introduction

Globally, a skilled public health workforce is essential for quality universal health coverage (UHC), which in turn contributes to improved population health (1). In disasters and crises, the public health workforce generates surveillance data with which to develop mitigation strategies and lead programs. The COVID-19 pandemic underscored the importance of public health leaders (2). Countries responded differently to the COVID-19 pandemic which, in part, was due to differing advice given by public health authorities and leaders (3). Public health professionals needed to advise despite limited evidence. Advisories given by public health experts about COVID-19, its transmission, prevention, and mitigation became controversial and were subject to political, media, and public scrutiny (4). Uncertainty resulted in the adoption of different prevention strategies such as lockdowns, in different countries. Uncertainties about therapeutics resulted in confusion (5) and misinformation. When preventive strategies became the focus, vaccine hesitancy (6) became prevalent, signifying mistrust of public health authorities and lack of confidence in their leadership. The aftermath of the pandemic has resulted in reflection about the need for leadership in pandemic preparedness (7) and the critical importance of leadership development (8).

The public health workforce and leaders must be grounded in a broad set of skills to effect Winslow’s seminal definition of public health (9)—“*the science and art of preventing disease … promoting health through the organized efforts of society …”* Having been trained in the broad range of disciplines—epidemiology, biostatistics, health promotion, environmental health, health economics, and health management—they need to apply these to societal health challenges to protect and promote the health of people. No single individual can have all competencies, and public health professionals and effective leaders need to operate within teams and know their limitations (10).

Leadership skills have long been identified as a core competency for Master of Public Health (MPH) courses in the United States (11). These include skills such as balancing the interests and needs of a multiplicity of stakeholders (11), working in and leading teams and being steeped in the ethos of social justice for decision-making, becoming ‘servant leaders’ (12). However, core leadership skills have not been a feature of postgraduate public health programs in other parts of the world (13), especially in sub-Saharan Africa.

In South Africa, the MPH qualification, introduced by 10 universities over the last three decades (14), is not a professional degree but is recognized as being the avenue for achieving competency in the field of public health practice. For most professionals who complete MPH programs, coursework is determined by university departments offering the degree which are accredited and quality assured by the mandated Council on Higher Education (15). There is no nationally agreed-upon set of public health competencies, course content, or graduate exit-level outcomes in the country. In addition, there is no country-wide association of schools of public health to guide or standardize these elements. In contrast, the European Association of Schools of Public Health (ASPHER) advocates for and updates its competencies for public health professionals (16). In the United States, the Association of Schools and Programs of Public Health (ASPPH) supports its member institutions through the accreditation process by the Council on Education for Public Health (CEPH), which ensures the quality of public health programs (17).

Prior research among University of KwaZulu-Natal (UKZN) MPH graduates found that they obtained report writing, critical analysis, research, leadership, and management skills (18). However, there has been limited educational research on MPH graduates from all South African MPH universities.

Consequently, a consortium of public health academics from eight South African universities conceptualized a large mixed methods study to begin to address these knowledge gaps. The broader study focused on the socio-demographic, educational, and occupational characteristics of the cohort of graduates, their motivations, career paths, perceptions of the curriculum, the utility of the degree, and its contribution to their professional and personal development. This study, which was nested in the broader research program, aimed to examine the motivations of the 2012–2016 cohort of MPH graduates for undertaking the MPH, the utility of the degree at work and in society and its contribution to their leadership skills. The study contributes to the discourse on curriculum reforms for leadership and the World Health Organization (WHO) articulated set of essential public health functions (EPHFs) (19).

## Methods

We identified all eight South African universities who graduated MPH students between 2012 and 2016 with academics from public health departments formed a consortium for this study. The institutions were Sefako Makgatho Health Sciences University (SMU), the University of Cape Town (UCT), UKZN, University of Limpopo (UL), University of Pretoria (UP), University of Venda (Univen), University of the Western Cape (UWC), and the University of the Witwatersrand (Wits). Alumni from SMU and UL were merged in the dataset as over this time these institutions became a single university—UL—with the same curriculum although teaching was at two campuses in different towns. Each university identified alumni’s names and, where possible, their contact details.

A cross-sectional online survey among alumni graduating between 2012 and 2016 from all eight South African universities was conducted. We had planned to conduct the research in 2020 but delays due to the COVID-19 pandemic meant that the study was executed between 2022 and 2023. The selection of the 2012–2016 cohort allowed for a minimum of 6 years of work experience post-qualification by which time alumni would have had time to settle into and reflect on their work. The survey consisted of closed-ended questions in the form of Likert scales and open-ended questions where respondents could comment freely.

### Development of the tool

Respondents’ demographic details were elicited through questions about their age, when they commenced the MPH, their sex, their prior training, current work roles, and country of origin. We probed their motivations for doing the MPH through asking them to rate the contribution of 10 factors to their decision-making using a five item Likert scale, where 1 = strongly disagree; 2 = disagree; 3 = neither agree nor disagree; 4 = agree; and 5 = strongly agree. There was space for free comments.

Based on Zwanikken et al.’s study (20), graduates’ self-perceived leadership skills were scored using five items for applicability and confidence in five leadership skills at work as 1 = not applicable; 2 = slightly applicable; 3 = moderately applicable; and 4 = very applicable. The extent these skills were obtained from the MPH were scored as 1 = not due to MPH; 2 = MPH enabled a little; 3 = MPH enabled moderately; and 4 = MPH enabled substantially. Their management of public health challenges at work was probed in the domains of management, academic, advocacy, and social responsiveness, using a 4 item Likert scale. The scoring system was 1 = not applicable; 2 = impact not due to MPH; 3 = MPH enabled a little; and 4 = MPH enabled substantially. In addition, alumni’s impact on society was probed through 9 items on a Likert scale used for impact at the workplace. The items scored are given in Table 1.

**Table 1:** Items scored for leadership and impact in the workplace, society and the MPH’s contribution.

**Leadership skills (applicable to work; obtained from the MPH)**	**Impact in workplace**				**Impact in society**
	**Management practice**	**Academic/Research**	**Advocacy at work**	**Social responsiveness**	
1. I can adapt my work environment or setting to cope with changes caused by public health emergencies such as the COVID-19 pandemic	1. I have created evidence (primary or secondary) for decision-making	1. I have developed a study or a research proposal.	1. I have published or posted in social media, with the intention of promoting health.	1. I have planned and implemented public health interventions, programs or policies based on consultation with stakeholders, using evidence and best practice.	1. I have contributed to changes in policy beyond my workplace.
2. I can apply team building skills in my workplace	2. I have reported and made recommendations on population health status or needs.	2. I have presented at conferences.	2. I have developed, reviewed or commissioned educational or Health Promotion media and materials.	2. I have developed, reviewed or I have implemented improvement strategies in response to findings arising from monitoring and evaluation.	2. I have contributed to changing guidelines, regulations or the law beyond my workplace.
3. I can apply conflict resolution skills in my workplace	3. I have contributed to change in policy in my workplace	3.I have published in peer reviewed publications	3. I have planned or implemented community health education courses or workshops.	3. I have contributed to improvements in human resource management.	3. I have contributed to influencing communities, organizations and/or sectors other than health.
4. I can describe the attributes of a good public health leader	4. I have contributed to change in policy at a level higher in the health system.	4. I have contributed to writing a published chapter of a book.	4. I have intervened or worked with the social determinants of health framework in a way that promotes equity.	4. I have contributed to improving working procedures, e.g. overcoming bureaucracy or inefficiencies.	4. I have contributed to equity/a pro-poor orientation in society.
5. I can apply human rights principles and equity in decision-making	5. I have participated in working committees focusing on program design or policy formulation at either provincial, national or international levels.	5. I raised a project grant.	5. I have collaborated/networked/developed partnerships successfully with departments other than health.	5. I helped initiate improvements within the workplace, or at another level in the health system.	5. I have contributed to changes in resource allocation for interventions; or conducted research, orientated towards equity and/or addressing the social determinants of health.
		6. I have participated in national or international collaborations.	6. I have collaborated with communities to initiate, sustain or evaluate projects.	6. I contributed to addressing the social determinants of health e.g. through planning processes, resource allocation or research.	6. I have promoted equitable access to services.
		7. I have tutored or taught public health professionals, trainees or students.			7. I have promoted access to quality services.
					8. I have contributed to increased resource mobilization for disadvantaged groups.
					9. I have influenced better understanding of public health measures in the general population.

### Recruitment

Eligible graduates needed to have graduated from one of the eight universities between 2012 and 2016, regardless of nationality or country of work. The survey was conducted between 2022 and 2023 after the COVID-19 pandemic. Each institution obtained a list of their graduates from their departmental student records. In two institutions, academic departments were not permitted to divulge the names and contact details of their graduates to the research team. Consequently, academics or administrators from these institutions invited graduates individually, by email, to participate in the online survey. A further institution invited graduates to participate through a notice sent out by the university central alumni office, following up with alumni by phone.

The level of detail of students’ academic exit dates and contact details varied by institution. This created obstacles to know confidently when some students graduated and whether eligible graduates had indeed been contacted. For most, a work and/or personal email and/or a mobile phone number were available. For five institutions, email details were available, and invitations to participate in the study were sent through the Research Electronic Data Capture (REDCap) which enabled tracking of non-responders. Automated reminders were sent to non-responders by REDCap.

**Figure 1:**
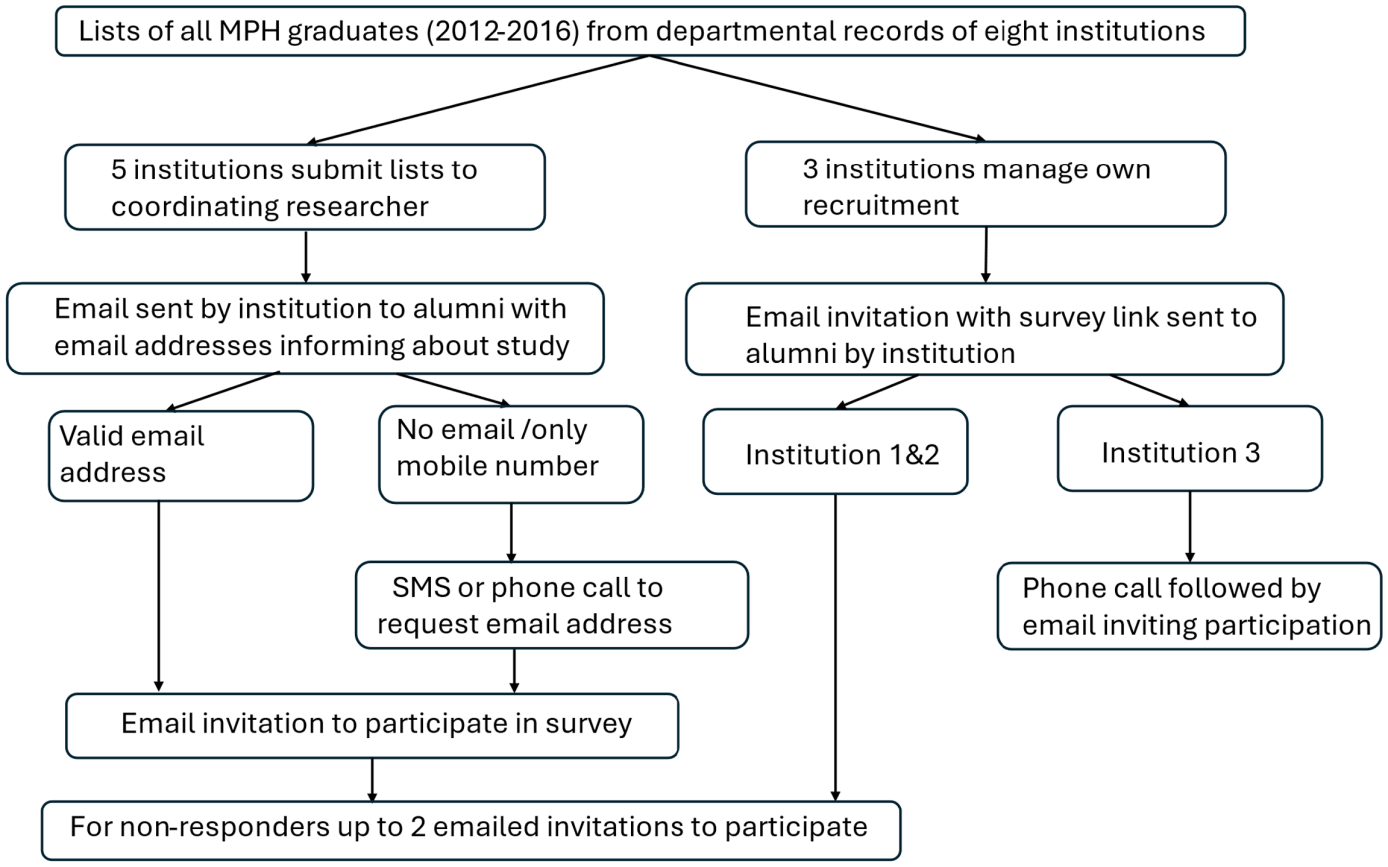
Recruitment strategy for MPH graduates.

If no email address was available but a mobile phone number was, mobile short messages (SMSs) were sent informing them that a study among MPH graduates from their institution was being conducted. Recipients were asked to reply to the sender and give a preferred valid email address should they be interested in the study. The initial recruitment did not yield a sufficient response and consequently where available, individualized personal email invitations were sent to non-responders and repeated twice. The text of the SMSs and telephone recruitment text is given in Supplementary file 1. The recruitment strategy is depicted in Figure 1.

The denominator for response rates was determined by counting confirmed current email addresses—alumni who we were confident had been successfully contacted. Participation in the survey required those invited to complete the online consent form before proceeding to complete the questionnaire.

Overall, there was a 35.5% response rate which varied by institution—from 18.1% to 70.3%, and 221 respondents completed the survey.

### Data storage and analysis

Online completion of the questionnaire facilitated encrypted data storage in REDCap. No identifiers were captured in the survey, and anonymized data were exported to STATA 13 for analysis. Data were cleaned by removing respondents who graduated before 2012 and those graduating after 2017. We combined the responses of alumni of SMU and UL as they were one institution from 2005 until April 2015 when they were unbundled, and course offerings were the same over the study period.

### Quantitative data analysis

Descriptive analyses were conducted for demographic, educational, and respondents’ roles for the time of the survey. Respondents’ ages, sex, and whether they were local or international students were compared to all MPH graduates between 2012 and 2016, using *χ^2^* tests for categorical data such as proportions, and the Mann–Whitney test for the non-normally distributed age data. Scores for motivations, leadership skills, and the impact of the MPH at work and in society were recoded. For motivations, ‘strongly disagree’ and ‘disagree’ were recoded to ‘disagree’; and ‘strongly agree’ and ‘agree’ to ‘agree’. Scores for confidence and applicability of leadership skills were recoded as 0 = not confident/applicable; 1 = slightly confident/applicable/MPH enabled a bit; 2 = moderately confident/applicable/MPH enabled a little; and 3 = very confident/applicable/MPH enabled substantially. Mean scores for leadership skills were calculated by summing each respondents’ score for the five items (given in Supplementary file 1), yielding a maximum score of 15 (5 items x 3). Alumni’s impact at work and the contribution of the MPH were also recoded. Not applicable to respondents was coded separately and enabled the calculation of the overall respondent proportions who exercised the skills.

The MPH’s contribution to respondents’ impact was recoded as 0 for ‘not at all’, 1 for ‘a little’, and 2 for ‘substantially’. The mean scores and 95% confidence intervals (CIs) of the contribution of the MPH for each impact item were calculated and converted to a percentage by dividing the mean, the lower, and upper values for the 95% CI by the maximum score for the number of respondents who contributed to the activity. Graphs for each item by domain are given in Supplementary file 2. We stratified the scores of respondents by prior qualification and job roles for leadership skills and impacts at work and in society which are given in Supplementary file 3. The stratum specific scores were compared to overall mean scores using the one sample *t*-test. Statistical significance is regarded as *p* ≤ 0.05.

### Qualitative data analysis

Free comments on motivations for completing the MPH were coded inductively, grouped into themes, and read in conjunction with scores. This gave deeper insight into what the scores were based on. Quotes that give substance and illustrate the scores are given. The country, age, and gender of respondents accompany the quotes.

### Ethical considerations

The study was first approved by the University of Cape Town’s Human Research Ethics Committee HREC: 630/2018 and then by participating institutions’ Ethics Committees, bar SMU which was combined with UL over the period graduates were enrolled in MPH programs. This process took time and delayed implementation. For the alumni of four institutions, potential respondents’ names were submitted to study collaborators responsible for recruitment, but for three, recruitment was through institutional academics as per Ethics Committees’ requirements. The identity of respondents is unknown as no identifiers were captured in the questionnaire unless respondents wished to be sent the findings of the survey. In these cases, the lists of emails supplied were removed from the dataset and stored separately. Prior to giving consent for participation, each respondent was given information about the survey, informed that they need not complete all sections or could withdraw with no consequence, and only once agreeing to participation were permitted to proceed. Investigators could not identify non-responders.

## Results

Respondents were alumni from seven universities who graduated MPHs between 2012 and 2016. From Table 2, this graduate cohort was a mid-career group of alumni, with a mean age of 35, who had a mean 8.2 (CI: 7.9–8.3) years’ work experience in public health post-MPH graduation. There were more women (57.5%) than men (41.6%). Most (53.2%) were South African, and less than 4% (3.5%; CI: 1.8–7.1%) came from outside Africa. Nearly two-thirds (65.4%; CI: 58.6–71.6%) had an undergraduate training in health. Post-graduation, respondents worked mostly as health service managers (40.7%; CI: 33.5–48.3%), in research or as academics (19,8%; CI: 14.4–26.5%), in health promotion (12.2%), or health policymaking (8.7%). Nearly one-third (31.0%) were employed by international or country-based non-governmental organizations (NGOs); over a quarter (27.0%) in government run health services; one-fifth (21,3%) by academic or research organizations; and 12.1% in hospital settlings. Respondents did not differ significantly from all MPH graduates from these universities over the same period by age, gender, or origin—South African compared to international students.

**Table 2:** Respondents demographics compared to all graduates, their educational backgrounds and roles.

**Respondents**		**All graduates**	**Comparing respondents to all graduates**
**Age (N=221)**	**Mean** **(95% CI)**	**Mean age** **(N=582; 95% CI)**	
Age at MPH commencement	35 (29-42)	37.0 (33.9-40.1)	z=-0.81; p=0.42
Age at survey	46 (41-53)		
**Gender (N=221)**	**Percentage (95%CI)**	**N=608**	
Male	41.6% (35.3-48.3%)	38.0% (34.2-41.9%)	z=-1.28; p=0.20
Female	57.5% (50.8-63.9%)	62.0% (58.1-65.7%)	
Other	0.9% (0.2-4.8%)		
**Country of origin (N=220)**		**N=605**	
South Africa	53.2% (46.5-59.7%)	56.0% (52.0-60.0%)	z=-0.16 p=0.87
Other African	43.2% (36.7-49.9%)		
**Prior profession (N=209)**			
Medical practitioner	20.7% (15.7-26.8%)		
Nursing	15.8% (11.5-21.5%)		
Other patient-facing health professionals	21.6% (16.5-27.8%)		
Other health trained	7.2% (4.3-11.7%)		
Natural Science trained	11.1% (7.4-16.2%)		
Humanities	10.2% (15.2-26.3%)		
**Employment (N=207)**			
Unemployed	8.2% (5.1-12.9%)		
Employed	91.8% (87.1-94.9%)		
Public health job (N=189)	92.1% (87.2-95.2%)		
**Role (n=172)**			
Program manager	27.3% (21.1-34.5%)		
Health service manager	13.4% (9.0-19.4%)		
Pure research	10.5% (6.7-16.1%)		
Health Promotion	12.2% (8.1-18.1%)		
Academic	9.3% (5.7-14.7%)		
Policy	8.7% (5.3-14.0%)		
**Employer (n=174)**			
Int & local NGO	31.0% (24.5-38.4%)		
National/provincial/district health managers	27.0% (20.9-34.1%)		
University/research institute	21.3% (15.8-28.0%)		
Public/private hospital	12.1 (8.0-17.9%)		
**Length of employment (n=173)**	**Years** **Median (IQR)**		
Employment post MPH	7 (6-9)		

### Motivations for studying

**Figure 2:**
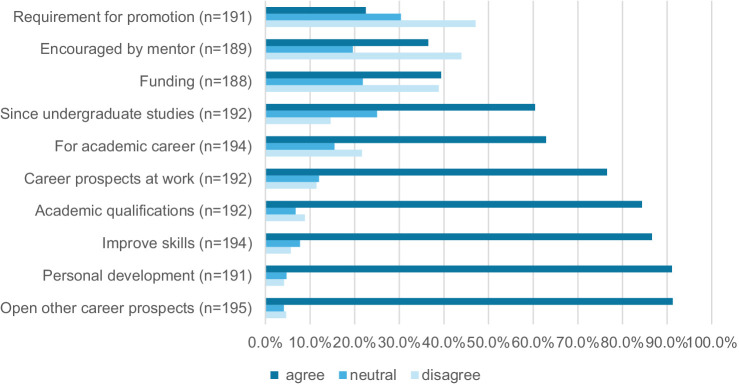
Motivations of MPH alumni for undertaking the MPH.

Respondents were motivated to complete the MPH for reasons that ranged from anticipated career benefits to enhancing their skills sets and for personal growth. Figure 2 shows that respondents identified that career prospects (91.3%; CI: 86.4–94.5%), personal development (91.1%; CI: 86.1–94.4%), an opportunity to improve work skills (86.6%; CI: 81.0–90.7%), obtaining a new academic qualification (84.4%; CI: 78.5–88.9%), and an improvement in career prospects in existing jobs (76.6%; CI: 70.0–82.1%) were important motivators for studying. Fewer were motivated by the availability of funding for fees (39.3%; CI: 32.6–46.6%), encouragement by a mentor (36.0%; CI: 29.4–43.1%), or that it was a requirement for promotion in their workplace (22.5%; CI: 17.1–29.0%).

Comments from respondents illuminate these motivators. For some, the MPH gave new public health perspectives to existing careers which enabled them to perform their work more effectively. For others, the MPH enabled a career change:

I couldn’t see myself progressing further with my degree in biomedical sciences. The vision before the MPH was patient specimen and results. But I saw that the same patients were coming back to hospital often with the same lab results. This did not make sense to me, and I knew that something else could be done to prevent reinfection. When I started the MPH, it became very clear to me that laboratory work could go very well with the new understanding of disease prevention and control. This kept me going. (Zambia, 50, F)

I wanted to change careers from being a clinician to rather working at the systems level (South Africa, 25,F)

For others, the MPH enabled personal growth and more “*confidence in the work*” (Ethiopia, 33, M) as they developed public health skills. This enabled them to advance their careers and take on leadership roles. It also gave graduates perspectives and skills to manage workplace challenges:

It prepares managers to counter the endless growing challenges in the health sector by gaining necessary skills to probe and analyze different issues that can impact the healthcare industry at any point in the future. (South Africa, 51, M)

Some emphasized that the added skills sets and academic qualifications were needed in their work context. Furthermore, others remarked that the skills learnt could open leadership positions in their work context.

[The] MPH degree … enables you to advance your career and take leadership roles in healthcare service delivery and people management as well as financial management. It gives you the professional foundation and skills you need to understand and work in a wide range of varieties of industries and fields. (South Africa, 51, M)

The work profile I was engaged in and doing required public health skills. To deliver my work effectively, I needed a formal degree as well. (India, 45, M)

In order to remain relevant and meaningfully employed in a middle to senior management or technical advisory role in the competitive NGO health sector, one has to have a tertiary qualification. (South Africa, 48, F)

The formal public health training and degree opened the possibility of academic careers, both teaching—“*to lecture at the university one day*” (Zambia, 33, F) and research careers which some desired. As one said, “*I have always wanted to become a public health researcher”* (Cameroun, 36, M).

Motivators that were not elicited through the questionnaire but were made in free comments were in the areas of health promotion, health literacy, and community development.

I was motivated to acquire knowledge and skills in public health, with a special interest for health promotion, prevention and restoration of public health of communities. (Namibia, 48, F)

I wanted to improve the health of communities through disseminating messages of health promotion, like washing hands before and after eating foods and after visiting the toilets, opening windows, having pit latrines for waste disposal, having small garden [s] for vegetables at homes and boiling drinking water from wells. (Malawi, 43, F)

In summary, their motivations centered on the acquisition of new and useful skills, for advancement at work. These could translate into their impact at work and in society.

### Leadership skills

The overall mean score for the use of the five leadership skills in Table 1 was 11.75/15 (s = 3.19) or 78.3% (CI: 72.5–82.1%) of the maximum score. Respondents’ confidence in being able to exercise their skills scored 81.3% (CI: 77.7–85.1%; x̅=12.3 s = 3.1) of the maximum, and these skills were rated as 50.7% (CI: 44.5–56.7%; x̅=7.60 s = 5.13) for being obtained from the MPH. Stratified by respondents’ prior qualification, and roles performed at work, those who obtained qualifications in non-patient-facing health degrees had significantly higher mean scores (x̅=14.0 s = 1.8) for leadership skills being applicable to their work (*t* = 3,5,898; *p* = 0.0089). Conversely, those working as pure researchers scored leadership skills significantly lower for being applicable to their work (x̅ =8.0; s = 4.11; *t* = 2,8,856; *p* = 0.018); for confidence in exercising them (x̅=9.2 s = 3.9; *t* = 2,473; *p* = 0.0356); and for being obtained from the MPH scored a mean of 25% (CI: 11.3–39.3%; x̅ =3.8 s = 3.36; *t* = −3.58; *p* = 0.006). In addition, those working as program managers were significantly more confident in the practice of leadership skills (x̅=13.4 s = 2.2; cf. 12.2 s = 3.1; *t* = 2,686; *p* = 0.013).

### Role of MPH for respondents’ impact at work and in society

Respondents’ impact at work explored in four domains: management practices, academic skills, advocacy, and being socially responsive are given in Table 3. As outlined below, activities were not applicable to all respondents, and this varied by specific prior qualifications and by roles at work (table in Supplementary file 3). In addition, activities performed were also rated by respondents as not being due to the MPH. Despite two exceptions in the academic domain (contributing a chapter to a book and raising a grant), over half the respondents rated that the MPH contributed to more than half of the activities in all domains.

**Table 3:** Role of the MPH in respondents’ impact at work and in society.

Item	Item description	n	Not applicable % (CI)	Not due to MPH % (95% CI)	Respondents identifying MPH as contributing to activity % (CI)	Mean score for MPH’s impact (CI)	Overall score percentage (mean score/total possible)
Impact on management at work							
1	Created evidence for decision-making	134	6.0 (3.0–11.6)	11.9 (7.4–18.7)	82.1 (74.6–87.8)	1.27 (01.15–1.39)	63.5 (57.5–69.4)
2	Reported on/made recommendations on population needs	133	12.8 (8.0–19.7)	6.8 (3.5–12.6)	80.4 (72.7–86.3)	1.17 (1.00–1.33)	58.4 (50.2–66.7)
3	Contributed to policy changes at work	134	17.2 (11.6–24.6)	13.4 (8.6–20.4)	69.4 (61.0–76.7)	0.98 (0.79–1.16)	48.8 (39.7–58.0)
4	Policy changes at higher level	134	17.2 (11.6–24.6)	11.9 (7.4–18.7)	70.9 (62.5–78.0)	0.93 (0.75–1.10)	46.4 (37.6–58.0)
5	Participation in high level committees	134	12.7 (8.0–19.6)	7.5 (4.0–13.4)	79.8 (72.1–85.9)	1.61 (1.39–1.83)	61.9 (53.5–70.3)
Academic impact at work							
1	Developed research proposal	130	4.6 (2.1–10.0)	6.2 (3.1–11.9)	89.1 (82.5–93.6)	1.48 (1.34–1.61)	73.8 (66.9–80.6)
2	Conference presentation	131	8.4 (4.7–14.6)	13.7 (8.8–20.9)	77.9 (69.8–84.2)	1.18 (1.02–1.34)	58.9 (50.9–67.0)
3	Published in peer–reviewed	126	26.2 (19.2–34.7)	19.8 (13.7–27.8)	54.0 (45.1–62.6)	0.64 (0.42–0.85)	31.8 (21.1–42.5)
4	Published chapter in book	129	55.0 (46.3–63.5)	15.5 (10.2–22.9)	29.5 (22.2–38.0)	–0.07 (−0.28–0.13)	–3.7 (−13.9–6.4)
5	Raised grant	130	34.6 (26.9–43.3)	25.4 (18.5–33.7)	40.0 (31.8–48.8)	0.34 (0.14–0.55)	17.2 (6.8–27.6)
6	Part of international collaboration	129	12.4 (7.7–19.4)	22.5 (16.0–30.6)	65.1 (56.4–72.9)	0.95 (0.77–1.13)	47.5 (38.5–56.5)
7	Taught students	131	30.5 (23.2–39.0)	13.7 (8.8–20.9)	55.8 (47.0–64.1)	0.60 (0.38–0.83)	30.1 (18.8–41.3)
Advocacy impact at work							
1	Used social media to promote health	131	23.7 (17.1–31.8)	19.1 (13.1–26.8)	57.2 (48.5–65.5)	0.66 (0.50–0.85)	33.1 (23.5–42.7)
2	Involved with develop of health promotion material	132	21.2 (15.0–29.1)	22.7 (16.3–30.8)	56.1 (47.4–64.4)	0.67 (0.46–0.87)	33.3 (23.1–43.6)
3	Involved in community education workshops	131	27.5 (20.4–35.9)	12.2 (7.6–19.1)	60.3 (51.6–68.4)	1.22 (1.07–1.37)	61.0% (53.5–68.5)
4	Worked with social determinants of health	129	27.5 (20.4–35.9)	12.2 (7.6–19.1)	65.1 (56.4–72.9)	0.74 (0.55–0.94)	37.2 (27.6–46.8)
5	Worked intersectionally	132	20.5 (14.3–28.3)	12.1 (7.5–19.0)	67.4 (58.9–75.0)	0.89 (0.70–1.08)	44.4 (34.9–53.9)
6	Engaged in community projects	132	22.7 (16.3–30.7)	12.9 (8.1–19.8)	64.4 (55.8–72.2)	0.73 (0.53–0.92)	36.3 (26.7–45.9)
Social responsiveness impact at work							
1	Developed Interventions based on consultation & evidence	130	10.0 (5.9–16.6)	10.0 (5.9–16.6)	80.0 (72.1–86.1)	1.11 (0.96–1.23)	55.7 (47.8–63.7)
2	Implemented improvement strategies	129	14.0 (8.9–21.2)	14.0 (8.9–21.2)	72.0 (63.6–79.2)	1.00 (0.82–1.18)	50.0 (41.2–58.8)
3	Improving human resources management	130	21.5 (15.2–29.5)	15.4 (10.1–22.8)	63.1 (54.4–71.0)	0.75 (0.56–0.94)	37.3 (27.8–46.8)
4	Overcome inefficiencies	128	23.1 (16.8–31.7)	16.2 (10.9–24.0)	60.7 (51.3–68.4)	0.75 (0.55–0.95)	37.5 (27.6–47.4)
5	Workplace improvements	130	19.5 (13.3–27.0)	14.8 (9.5–21.9)	65.7 (57.5–73.9)	0.93 (0.74–1.12)	46.3 (36.8–55.8)
6	Addressed social determinants of health	130	12.3 (7.6–19.3)	10.8 (6.4–17.5)	76.9 (68.8–83.4)	1.17 (1.00–1.34)	58.6 (49.9–67.3)
Impact on society							
1	Changes beyond workplace	129	23.3 (16.7–31.4)	15.5 (10.2–22.9)	61.2 (52.5–69.3)	0.68 (0.49–0.87)	33.9 (24.3–43.5)
2	Change to regulations/law beyond workplace	129	24.8 (18.0–33.1)	17.1 (11.4–24.7)	58.1 (49.3–66.4)	0.67 (0.47–0.87)	33.5 (23.5–43.4)
3	Participated in organizations outside of health	130	19.2 (13.3–27.0)	18.5 (12.6–26.2)	62.3 (53.6–70.3)	0.78 (0.59–0.97)	38.9 (29.5–48.4)
4	Contributed to a pro–poor society	130	23.1 (15.6–21.2)	20.0 (13.9–27.9)	56.9 (48.2–65.3)	0.63 (0.44–0.82)	31.6 (22.1–41.0)
5	Contributed to societal equity interventions	128	23.4 (16.8–31.7)	11.7 (7.1–18.6)	64.9 (56.1–72.7)	0.73 (0.54–0.93)	36.7 (27.0–46.3)
6	Promoted equitable service access	130	20.0 (13.9–27.8)	13.8 (8.8–21.0)	66.2 (57.5–73.9)	0.79 (0.60–0.98)	39.3 (29.9–48.8)
7	Promoted access to quality services	128	16.4 (10.9–24.0)	14.8 (9.6–222.2)	68.8 (60.1–76.3)	0.88 (0.70–1.07)	44.2 (35.0–53.3)
8	Contributed to resource mobilization for disadvantaged	130	23.1 (16.6–31.2)	13.1 (8.2–20.1)	63.8 (55.1–71.7)	0.79 (0.59–0.98)	39.3 (29.5–49.2)
9	Promote understanding of public health in society	129	13.2 (8.2–20.3)	12.4 (7.7–19.4)	74.4 (66.1–81.3)	1.03 (0.86–1.21)	51.6 (42.9–60.4)

#### Management practices at work—contributions to policy and program development

In this domain, between 69.4% (CI: 61.0–76.7%) and 82.1% (CI: 74.6–87.8%) of respondents contributed to policy changes at work and created evidence for decision-making, respectively. However, respondent scores of the MPH’s contribution to their impact were low and varied between 46.4% for effecting policy changes at a higher level to 63.5% for creating evidence for decision-making.

There were no differences in the proportions of respondents who did not participate in the named activities by educational background and roles at work with one exception. Significantly lower proportions of those working as program managers rated that they did not make contributions to policymaking at higher levels in the health system (2.1%; CI: 0.3–14.0% cf. 9.2%; CI: 6.1–13.4%; z = 13.6; *p* = 0.00).

There were no significant differences in the scoring of the MPH’s contribution to respondents’ impact in policy and program development by educational background or current roles. However, respondents with non-patient-facing health training scored the MPH higher for the MPH assisting them in creating evidence for decision-making (x̅=1.86; s = 0.38 cf. x̅=1.27; s = 0.67; *t* = 4.11; *p* = 0.0063) and contributing to policy changes at higher levels of the health system (x̅=1.83; s = 0.41 cf. x̅=1.23; s = 0.69; *t* = 3.62; *p* = 0.0152). In addition, respondents, with roles in health promotion, scored the MPH significantly higher for enabling them to make recommendations on populations needs (x̅=1.77; s = 0.44 cf. x̅=1.41; s = 0.63; *t* = 2.95; *p* = 0.0121) and their participation in high level committees (x̅=1.80; s = 0.41 cf. x̅=1.47; s = 0,65; *t* = 3.087; *p* = 0.008).

#### Academic/research

Academic contributions were not relevant for a wide proportion of respondents. More than a half (55.0%) had not published a chapter in a book and roughly a third (34.6%) had never raised a grant or taught students (30.5%). Nonetheless, a high proportion (95.5%; CI: 90–97.9%) had developed a research proposal, for which they scored the MPH as making a substantial contribution (73.8%). Most (91.6%; CI: 85.4–95.3%) had presented at conferences, but they rated that the MPH contributed only 58.9% to this activity. A large proportion participated in international collaborations (87.6%; 80.6–92.3%), where however, the MPH contributed less than 50% (47.5%; CI: 38.5–56.5%). Nearly three-quarters (73.8%; CI: 65.3–80.8%) had published in peer-reviewed publications, but again the MPHs contributed to less than a third (31.8%) to this activity. The MPH was scored as contributing less than 50% to other respondents’ other academic impacts.

#### Advocacy at work

The various activities listed under advocacy impacts at work were relevant for large proportions of respondents. Approximately three-quarters of respondents—76.3% (CI: 68–82.9%), worked to address social determinants of health, and 79.9% (CI: 71.7–85.7%) of respondents worked intersectionally. However, less than 70% rated the MPH as contributing to their impacts, and on average the MPH scored less than 50% for these activities except for being involved in community education activities where it scored 61.0% for the 72.58% (CI: 64.0–79.6%) who participated in community education activities.

Working intersectionally was stratified by prior education and roles. There were significantly higher proportions of those with a pure science background (39.1%; CI: 12.3–60.3%) and those working as researchers (27.8%; CI: 11.7–52.8%) who did not work intersectionally. Conversely, significantly smaller proportions of those working as health service managers did not work intersectionally (4.3%; CI: 0.6–37.2%) cf. 10.8% (CI: 7.5–15.3%). Those working in the policy field scored the MPH significantly higher for their ability to work intersectionally (x̅=1.73; s = 0.47; cf. x̅=1.27 s = 0.71 overall; *t* = 3.247; *p* = 0.009). Conversely, those working as health service managers (x̅=0.91 s = 0.30 cf. x̅=1.27 s = 0.71; *t* = −3.97; *p* = 0.0026) or in pure research (x̅=0.86; s = 0.38) scored the MPH significantly lower in its contribution to their ability to work intersectionally.

#### Social responsiveness

The six identified skills related to social responsiveness were reported as relevant by between 76.6% (CI: 68.3–83.2%) and 90.0% (CI: 83.4–94.1%) of respondents. The lowest relevance attributed was to overcoming inefficiencies (76.6%) and the highest to developing evidence-based interventions (90.0%). When asked about the MPH degree’s contribution to these impacts, between 60.7% (CI: 51.3–68.4%) and 80.0% (CI: 72.1–86.1%) of respondents acknowledged its influence, again lowest for overcoming inefficiencies and highest for developing evidence-based interventions. For two of the six skills, addressing social determinants of health (58.6%) and developing evidence-based interventions, more than half of the respondents attributed over 50% of their impact to MPH training. The remaining four skills scored below 50% in terms of the MPH’s contribution to respondents’ perceived impact.

Significantly higher proportions of those with a pure science background (between 21.7% and 34.8% for four of the six skills) and those working in pure research (between 22.8% and 33.3% for five of the six activities) rated that they did not participate in the identified social responsiveness activities. In addition, a significantly higher proportion of those with non-patient facing health training did not participate in evidence based interventions (20%; CI: 6.3–48.3% cf. 5.2%; CI: 3.0-7.7%; z = 2.47; *p* = 0.014) or human resources management (26.7%; CI: 9.9–54.5% cf. 11.2%; CI: 7.8–15.7; z = 2.64; *p* = 0.008). On the other hand, significantly larger proportions of program managers contributed to overcoming inefficiencies at work (97.9%; CI: 62.8–99.7% cf. 88.0%; CI: 83.4–93.2%; z = 2.01; *p* = 0.0438).

The only differences in scores by prior education and roles were for developing evidence-based interventions. Respondents with prior training as a nurse (x̅=1.47 s = 0.62 cf. x̅=1.05 s = 0.92; *t* = 2,7,778; *p* = 0.0134) and those working in health promotion (x̅= 1.67 s = 0.49 cf. x̅=1.05 s = 0.92; *t* = 4.895; *p* = 0.0002) rated that the MPH contributed significantly more to this activity.

#### Societal impact

More than three quarters of respondents affirmed that they were involved in all the given activities that had impact in society. The highest score for the impact of the MPH on activities was promoting an understanding of public health in society. Of the 86.8% (CI: 79.7–91.7%) who reported that they promoted an understanding of public health in society, 74.4% rated the MPH as playing a role; yet the MPH contributed to 51.6% of their impact. While 76.9% (CI: 68.8–83.4%) agreed that they contributed to a pro-poor society and 75.2% (CI: 66.9–82.0%) contributed to changes in laws/regulations beyond the workplace, only 56.9% and 58.1%, respectively, rated the MPH as having impact, which was the only item that scored less than 60% for the impact of the MPH.

Significantly higher proportions—between 21.7% (CI: 9.1–43.5%) and 30.4% (CI: 14.9–52.2%) of respondents (*p* < 0.05)—with pure science training prior to the MPH rated that they did not have impact in seven of the nine items [changes in society (30.4% cf. 12.0% z = 2.30 *p* = 0.02); in laws/regulations beyond the workplace (30.4% cf. 12.7% z = 2.47; *p* = 0.014); in interventions that promote social equity (34.8% cf. 11.9% z = 2.24; *p* = 0.025); in promoting access to quality services (26.1% cf. 10.4%; z = 2.30; *p* = 0.021); mobilizing resources for the disadvantaged (30.4% cf. 12.0% z = 2,47; *p* = 0.014); and promoting an understanding of public health in society (21.7% cf. 6.8%; z = 2.52; *p* = 0.012)]. A significantly larger proportion of those with non-patient facing health degrees also did not promote access to quality health services (26.7% cf. 8.4% z = 2.36; *p* = 0.019). In addition, significantly smaller proportions of those working as pure researchers rated that they contributed to a pro-poor society (66.7% cf. 88.0%; z = 2.52; *p* = 0.012); participated in organizations outside of health (72.2% cf. 90.1% z = 2.3; *p* = 0.02); and promoted an understanding of public health in society (77.8% cf. 93.2% z = 2.36; *p* = 0.018). In addition, smaller proportions of those working in health promotion (69.6% cf. 88.0%) contributed to mobilizing resources for the disadvantaged. Conversely, all those with humanities degrees affirmed that they contributed to interventions in society that promoted equity (100.0% cf. 88.1%, z = 2.24; *p* = 0,025).

Those with pure science backgrounds scored the MPH significantly higher for enabling them to effect changes beyond the workplace (x̅=1.67; s = 0.52 cf. x̅=1.12; s = 0.72 t = 2.593; *p* = 0.049), whereas those with a humanities background scored the MPH significantly lower (x̅=0.87; s = 0.52 t = 2.20; *p* = 0.045). Those working in health promotion scored the MPH higher than all respondents their contribution to a pro-poor society (x̅=1.58 s = 0.67 cf. x̅=1.03 s = 0.74 t = 2.867; *p* = 0.015) and their promotion of access to quality services (x̅=1.62 s = 0.51 cf. x̅=1.18 s = 0.71; t = 3.100 *p* = 0.009). Furthermore, academics scored the MPH significantly higher, enabling their contribution to the understanding of public health in society (x̅=1.67 s = 0.50 cf. x̅=1.28 s = 0.70). Conversely, those with humanities degrees scored the MPH significantly lower (x̅=0.87 s = 0.52 cf. x̅=1.16 t = 2.20; *p* = 0.045) for their contribution to interventions promoting social equity.

## Discussion

This research, the first known from South African and African universities with MPH programs, explored graduates’ motivations for undertaking the MPH, their leadership skills and their perceived impact of public health skills in their workplace in four domains—management, academic, advocacy, and social responsiveness as well as their impact in society, and the contribution of the MPH to these (20).

This set of graduates, mostly in their mid-30s from sub-Saharan Africa, with undergraduate training largely as health professionals, was motivated to complete the MPH to improve their career prospects and skills sets, promoting their personal development for senior management and leadership roles. They took on roles as health managers—both in program and line management, in research and as academics, and in health promotion and policy work. These findings are similar to two South African studies that explored the motivations of applicants for MPH studies (21) and a graduate survey of doctors who completed an MPH (22). The former highlighted that applicants wanted to do an MPH to acquire knowledge about public health promotion and prevention, acquiring skills to become public health practitioners, and the latter found that they wanted to transition from clinical to management or academic work, believing that the MPH opened career opportunities.

The focus on developing skills that enabled career advancement highlighted in this study may be due to this cohort of graduates having on average nine pre-MPH work years and a mean of eight post-MPH work experience, a total of 17 years, giving them time for reflection, interpreting the meaning and role of the MPH in their careers.

Overwhelmingly, respondents were confident using most leadership skills (81%) which were required at work (78%). Yet, just half were obtained through the MPH, implying that leadership skills needed to be obtained elsewhere. Indeed, following the COVID-19 pandemic, a review of accredited MPH programs in the United States found that leadership training was found to be sorely lacking (23) and the importance of leadership competency has been highlighted for European programs (24). In this study, the only group who scored differently worked as researchers who scored leadership skills significantly lower by their confidence to exercise these, being needed at work, and being obtained from the MPH.

Motivations for undertaking the MPH were congruent with their self-assessed impacts and over three-quarters of alumni were involved in activities in public health domains such as management—particularly in policy development, in academic functions, advocacy, and social responsiveness at work and in society. Nonetheless, some academic activities such as publishing in a book (relevant for 45%), raising a grant (relevant for 66%) and teaching (relevant for 70%) scored low. These activities are primarily undertaken by senior academics, so it is not surprising that many had not engaged in them or rated that the MPH contributed substantially to these. Nonetheless, research skills, such as proposal writing, were used by close to 90% of respondents who rated the MPH as having a large contribution (78%) to these skills.

Overall, the MPH contributed to respondents’ impact in all domains. Some skills learnt in the MPH were rated highly, such as working in high level committees which suggests alumni’s leadership roles. There were some differences among respondents by educational background and roles at work, particularly those who had a pure science background or worked as researchers. Significantly larger proportions of these respondents rated that they did not perform many activities associated with social responsiveness at work or impact in society, suggesting that larger proportions of these respondents performed defined technical roles. However, those scientists involved in these activities scored the MPH’s contribution significantly higher to their ability to make contributions beyond the workplace. Conversely, researchers scored the MPH lower in enabling them to work intersectionally.

Leadership skills probed and those impacting on workplaces include those that are core to respondents’ job descriptions as well as activities initiated independently. For example, program managers were more confident using leadership skills suggesting that their work required them to lead teams and make decisions.

Contributions in the four impact domains also depended on the jobs they assumed. For example, only 20% were employed as academics and researchers, and consequently, it is not surprising that high level academic roles such as grant writing and book publishing were important for a minority. While research skills are core to the MPH and producing a research report is required for the degree., the majority presented at conferences which suggests personal agency and confidence.

Roles listed under management practices demonstrate that most graduates participated in decision-making, contributed to generating evidence for policy and decision-makers. They participated in high level committees and policymaking—evidence for their senior managerial roles. Those with backgrounds in non-patient-facing health training and those working in health promotion scored the MPH higher for enabling them to create evidence for decision-making/policymaking at a higher level and making recommendations on population needs/participation in high level committees, respectively. While the majority took on advocacy roles, two-thirds of the specified items are specific to job-descriptions such as health promotion and two—working intersectionally and with social determinants of health—could span a range of managerial or technical roles. These latter two complex roles, involving a range of stakeholders, require social strategy and interpersonal skills, a hallmark of public health leaders (11).

Most impacts, given under social responsiveness, speak to agency to improve work environments—working with teams to create environments conducive to productive work. Their impacts in society point to their agency to effect changes in the broader society. Again, close on 80% rated that they were change agents beyond their workplace—participating in other organizations and interventions toward social justice and equity, values that are core to ‘servant leadership’ (12). Some activities such as promoting an understanding of public health in society and changes to regulations indicate that they took on innovative roles at a high level in organizations, as leaders.

The MPH in South Africa was initially intended to produce a workforce to improve health status and healthcare in the region (25). While universities offer a heterogenous range of MPH tracks, most require completion of core courses that include public health foundational domains such as epidemiology, health systems and policy, and social and behavioral sciences. Some programs explicitly intend to prepare professionals to take on leadership roles (26–28), and others focus on acquiring skills to make a contribution to health systems (29, 30). Some offer health management courses, but none offer dedicated courses focused on leadership. Instead, some university public or global health departments offer post-graduate diplomas in health management and leadership believing that health leaders require exposure to a range of management skills and experiential learning to enable the navigation of complex health systems (31–33).

Nonetheless, graduates participated in policy processes and contributed to social responsibility, which implies an engagement in leadership roles. Their roles in complex work and societal settings indicate that these skills were acquired despite not being explicitly taught in the MPH. Many may have been acquired elsewhere and experientially over time, which is possible given that graduates had approximately 16 years’ work experience prior to completing the survey.

### Limitations

Despite concerted institutional effort to encourage participation, this national survey of alumni from all South African universities with MPH graduates between 2012 and 2016 achieved a 35.5% response rate. The low response rate does not mean that a selection bias was present as respondents resembled the age and gender of the source population of MPH graduates. Despite this, respondents may be more positive about the MPH and the competencies they acquired than non-responders. Analyses demonstrated few differences by strata (prior training and job roles) for leadership skills and impacts, and confidence intervals were wide. This implies that the sample was underpowered and too small to detect differences where there may exist, a type 2 error.

Leadership skills assessed and the impact framework used was developed by other researchers in LMICs (20). However, we did not invite free comments or examples of graduates’ leadership roles and impact. This limited obtaining more in-depth information and examples about graduates’ roles. The tool also did not enquire about specific leadership roles, which would have assisted in understanding the extent and nature of their leadership at work and in society, including their roles as decision-makers. A further limitation was that we did not enquire where in the MPH programs they acquired competencies for these impacts. Free comments were, however, invited for their motivations which gave in-depth information about their intentions for completing an MPH.

## Conclusion and recommendations

This mid-career cohort of MPH graduates reported that they used leadership skills and had an impact at work and in society which was in part due to skills obtained through the MPH. Public health professionals require a keen analysis of context-specific disease burdens, their determinants, and competencies to determine how best to manage these in the short, medium, and longer term. This requires a recognition of one’s limitations (11), of other team members’ strengths and an appreciation of the importance of transparent processes. In addition, in crises, evidenced by the COVID-19 pandemic, leaders require excellent communication, collaboration, and critical decision-amidst considerations of social justice and equity (34). Health system leaders and decision-makers require astute health managers and public health trained experts to weigh in and advise. These experts, in turn, must be supported by technical staff who can generate information from a range of sources.

While public leadership skills are not the focus of many MPH programs in South Africa, they are clearly needed. In view of the growing number of MPH programs and the increasing numbers of graduates who, through the MPH, intend advancing careers, MPH programs should place a greater emphasis on preparing MPH graduates for leading teams and playing key roles in health systems leadership, which is echoed elsewhere (23, 24). These skills need to be purposefully introduced and could be integrated into MPH courses through, for example, students reflecting on their experiences and roles in projects through both formative and summative assessments.

MPH program reviews are timely and need to adapt to a post-COVID-19 world. Given that the MPH is largely the degree for public health work in LMICs, programs need to produce a workforce that is ‘fit for purpose’. This requires an alignment of education objectives with workforce systems in country contexts so that the transition between being a learner and worker is seamless (19). In some Asian country settings, there is advocacy for public health educational frameworks to include communication, leadership, and management skills as well as analytical, policy, and planning skills for country settings (35–37). In South Africa, consideration needs to be given for the formation of an association of a national schools of public health, who can engage with the range of employers about required competencies.

Such initiatives align with the ambitious World Health Organization (WHO) roadmap project, which, responding to the dearth of public health personnel globally, aims to embed the public health workforce (38) in country health systems (39). Their detailed project report (19) identified 20 competencies in six domains—community-centeredness, decision-making, communication, collaboration, evidence-informed practice, and personal conduct for public health practice, which are patently the skill sets of health leaders. The translation and application of the WHO Roadmap project into country health systems requires bold leadership; an appreciation of country-specific disease burdens and health systems; and maps of diverse public health actors. This must comprise the full range of public health professionals, their professional organizations, training organizations, employers, and certifying institutions (40). This project requires trusting intentional collaboration among this range of actors who need to clearly articulate shared goals. They must be credible public health leaders, who develop strategies and transparently operationalize plans to reform educational programs and robust health systems able to deliver essential public health functions.

## Data Availability

The datasets presented in this article are not readily available because consent for sharing of data was not obtained from participants in this study. Requests to access the datasets should be directed to virginia.zweigenthal@uct.ac.za.
