# Supplementary material for: Perceptions of South Africa’s master of public health graduates on the degree’s contribution to their leadership at work and in society
Source: Front Public Health. 2025 Oct 8;13:1620477. doi: 10.3389/fpubh.2025.1620477 (PMC12540475; doi:10.3389/fpubh.2025.1620477)
Supplement: Supplementary file 1 [file Data_Sheet_1.docx]

Questionnaire used in survey:

1. Demographic information
2. To determine motivations for MPH studies
3. Leadership skills
4. The utility of the MPH at work and
5. In society

AND

1. Texts used to promote recruitment
2. Demographic information
   1. Sex Female/Male/Prefer not to answer
   2. Age __________
   3. Which country are you from? (drop down list) ________________________
   4. Years of work experience before you started your MPH ___
   5. Prior degree ________
   6. Length of employment since MPH (for public health employed) ______________
   7. Current work
      1. What best describes your most recent place of employment?
         1. National Ministry of Health
         2. Regional/Provincial Health Department
         3. District Health Department
         4. Public hospital or Clinic
         5. College/university
         6. Research institute
         7. International NGO
         8. Local NGO
         9. Private hospital/clinic
         10. Private for profit organization
         11. Self employed
         12. Other: what: ____________
      2. Which of the following best describes your work responsibilities at your most recent workplace?
   8. Clinical care/service for individual patients
   9. Health Promotion (including health education, disease prevention or control, communication, social marketing
   10. Academic in tertiary education institution
   11. Pure research
   12. Information management
   13. Health service (line) management
   14. Program/project management
   15. Public communication and involvement with stakeholders
   16. Policy process
   17. Student
   18. Other: What: _______________
3. Leadership skills

| **Applicability of skill to current or most recent job (5 items below) Max score: 15** | | |  | **Confidence in possession of skill (5 items below Max score: 15** | | |  | **Extent to which MPH enabled acquisition of skill (5 items below) Max score: 15** | | |
| --- | --- | --- | --- | --- | --- | --- | --- | --- | --- | --- |
| **Option** | **Score** | **Rescored** |  | **Option** | **Score** | **Rescored** |  | **Option** | **Score** | **Rescored** |
| Not applicable | 1 | 0 |  | Not confident | 1 | 0 |  | Not due to MPH | 1 | 0 |
| Slightly applicable | 2 | 1 |  | A little confident | 2 | 1 |  | MPH enabled a little | 2 | 1 |
| Moderately applicable | 3 | 2 |  | Moderately confident | 3 | 2 |  | MPH enabled moderately | 3 | 2 |
| Very Applicable | 4 | 3 |  | Very confident | 4 | 3 |  | MPH enabled substantially | 4 | 3 |

1. I can adapt my work environment or setting to cope with changes caused by public health emergencies such as the COVID-19 pandemic
2. I can apply team building skills in my workplace
3. I can apply conflict resolution skills in my workplace
4. I can describe the attributes of a good public health leader
5. I can apply human rights principles and equity in decision-making
6. Motivation for MPH studies

| Question | Strongly disagree | Disagree | Neither agree nor disagree | Agree | Strongly agree |
| --- | --- | --- | --- | --- | --- |
| My undergraduate studies piqued my interest in public health |  |  |  |  |  |
| The MPH was a requirement for promotion at work |  |  |  |  |  |
| The MPH is a stepping stone to an academic or research career |  |  |  |  |  |
| The MPH could improve my career prospects at my place of work |  |  |  |  |  |
| The MPH could improve my career prospects outside my place of work (e.g. it could open up other career paths/options |  |  |  |  |  |
| The MPH would upgrade my academic qualifications |  |  |  |  |  |
| Funding was available |  |  |  |  |  |
| My mentor encouraged me to pursue an MPH |  |  |  |  |  |
| The MPH could lead to personal development |  |  |  |  |  |
| Any other motivations for completing the MPH | Yes | | No | | Give detail |

1. IMPACT IN YOUR WORKPLACE

This section asks questions about how you see the MPH enabled you to impact on your workplace (Depending on your work or the context of your work or workplace, it is possible that not all sections and statements apply to you.).

[***NB: these scores were recoded as follows:***

***Score: Not due to MPH 0***

***A little due to MPH 1***

***MPH enabled substantially 2]***

**MANAGEMENT PRACTICE**

|  |  | **1 Not applicable to me** | **2 My impact is not attributable to the MPH** | **3 The MPH enabled me a little to impact** | 1. **The MPH enabled me substantially to impact** |
| --- | --- | --- | --- | --- | --- |
| 1 | I have created evidence (primary or secondary) for decision-making. |  |  |  |  |
| 2 | I have reported and made recommendations on population health status or needs. |  |  |  |  |
| 3 | I have contributed to change in policy in my workplace. |  |  |  |  |
| 4 | have contributed to change in policy at a level higher in the health system. |  |  |  |  |
| 5 | I have participated in working committees focusing on program design or policy formulation at either provincial, national or international levels. |  |  |  |  |

**ACADEMIC/RESEARCH**

|  | **SCALE** | **1 Not applicable to me** | **2 My impact is not attributable to the MPH** | **3 The MPH enabled me a little to impact** | **4 The MPH enabled me substantially to impact** |
| --- | --- | --- | --- | --- | --- |
| 1 | I have developed a study or a research proposal. |  |  |  |  |
| 2 | I have presented at conferences. |  |  |  |  |
| 3 | I have published in peer reviewed publications. |  |  |  |  |
| 4 | I have contributed to writing a published chapter of a book. |  |  |  |  |
| 5 | I raised a project grant. |  |  |  |  |
| 6 | I have participated in national or international collaborations. |  |  |  |  |
| 7 | I have tutored or taught public health professionals, trainees or students. |  |  |  |  |

**ADVOCACY AT WORK**

|  | **SCALE** | **1 Not applicable to me** | **2 My impact is not attributable to the MPH** | **3 The MPH enabled me a little to impact** | 1. **The MPH enabled me substantially to impact** |
| --- | --- | --- | --- | --- | --- |
| 1 | I have published or posted in social media, with the intention of promoting health. |  |  |  |  |
| 2 | I have developed, reviewed or commissioned educational or Health Promotion media and materials. |  |  |  |  |
| 3 | I have planned or implemented community health education courses or workshops. |  |  |  |  |
| 4 | I have intervened or worked with the social determinants of health framework in a way that promotes equity. |  |  |  |  |
| 5 | I have collaborated/networked/developed partnerships successfully with departments other than health. |  |  |  |  |
| 6 | I have collaborated with communities to initiate, sustain or evaluate projects. |  |  |  |  |

**SOCIAL RESPONSIVENESS**

|  | **SCALE** | **1 Not applicable to me** | **2 My impact is not attributable to the MPH** | **3 The MPH enabled me a little to impact** | 1. **The MPH enabled me substantially to impact** |
| --- | --- | --- | --- | --- | --- |
| 1 | I have planned and implemented public health interventions, programs or policies based on consultation with stakeholders, using evidence and best practice. |  |  |  |  |
| 2 | I have developed, reviewed or I have implemented improvement strategies in response to findings arising from monitoring and evaluation. |  |  |  |  |
| 3 | I have contributed to improvements in human resource management. |  |  |  |  |

| 4 | I have contributed to improving working procedures, e.g. overcoming bureaucracy or inefficiencies. |  |  |  |  |
| --- | --- | --- | --- | --- | --- |
| 5 | I helped initiate improvements within the workplace, or at another level in the health system. |  |  |  |  |
| 6 | I contributed to addressing the social determinants of health e.g. through planning processes, resource allocation or research. |  |  |  |  |

1. IMPACT IN SOCIETY

This section asks questions about how you see the MPH enabled you to impact on your workplace.

(Depending on your work or the context of your work or workplace, it is possible that not all sections and statements apply to you.)

|  | **SCALE** | 1. **Not applicable to me** | 1. **My impact is not attributable to the MPH** | 1. **The MPH enabled me a little to impact** | 1. **The MPH enabled me substantially to impact** |
| --- | --- | --- | --- | --- | --- |
| 1 | I have contributed to changes in policy beyond my workplace. |  |  |  |  |
| 2 | I have contributed to changing guidelines, regulations or the law beyond my workplace. |  |  |  |  |
| 3 | I have contributed to influencing communities, organizations and/or sectors other than health. |  |  |  |  |
| 4 | I have contributed to equity/a pro-poor orientation in society. |  |  |  |  |
| 5 | have contributed to changes in resource allocation for interventions; or conducted research, orientated towards equity and/or addressing the social determinants of health. |  |  |  |  |
| 6 | I have promoted equitable access to services. |  |  |  |  |
| 7 | I have promoted access to quality services. |  |  |  |  |
| 8 | I have contributed to increased resource mobilization for disadvantaged groups. |  |  |  |  |
| 9 | I have influenced better understanding of public health measures in the general population. |  |  |  |  |

1. TEXTS USED TO PROMOTE RECRUITMENT
2. **SMS to potential respondents with known phone numbers**

Hi [potential respondent NAME]. [NAME] from [Institution name] here. SA universities graduating MPH students are conducting research among alumni who completed their degree from 2012 to 2016 to find out its impact and usefulness. It would be wonderful if you could complete it. Our response rate isn't good enough to have solid findings and recommendations I don't have a an e-mail address for you. (It is an online questionnaire best completed on a PC.) Can you send me a good e-mail address to send the invitation to you.

1. **Script for telephone calls to potential respondents**

**Good day**

Please can I speak to …. (need to confirm that you are speaking to the correct person)

My name is [] from [Institution]. Is this a convenient time for you to speak?

1. No
2. Yes

If the answer is “No”, you should say: **When will be a convenient time for you?**

Remember to call back.

**If yes, or at the call back**

I am assisting []. We are conducting an MPH Graduate Survey together with seven other South African universities: [list]

The survey aims to obtain the perspectives of all graduates from these universities for the period 2012-2016 on the utility (or value) of the MPH degree and the effect it has had on their career trajectories.

We do not have your email address and would like to send you the link to the survey. Would you mind giving me your email address?

***What do you want to do with my email address?***

We would like to send you the link to the survey (Repeat information if necessary).

***Why are you doing the survey?***

The survey aims to obtain the perspectives of all graduates from 8 universities for the period 2012-2016 on the utility (or value) of the MPH degree and the effect it has had on their career trajectories.

As you know, the Covid-19 pandemic has underscored the importance of competent public health professionals and practitioners. Hence, we want to use the survey results to recommend or promote career paths for MPH graduates and to contribute to curricular reforms (if necessary).

***What if I do not want to take part in the study?***

Participation in this study is voluntary. If the response rate is low, the results will be biased because the perspectives of those that respond may be different to those that have not responded. This means that we will not be able to influence government or global policy for the benefit of our MPH graduates.

**Hopefully, the person will give you the email address:**

1. Make sure that you write down the email address.
2. Confirm with the person by reading it back.
3. Call each person at least 3 times before giving up.
4. Note if wrong number.
5. Note down if the person refuses to give you the email address or say that they are not interested in the study.

**Thank the person for taking the call and for giving you the email address!**
