# Supplementary material for: Perceptions of South Africa’s master of public health graduates on the degree’s contribution to their leadership at work and in society
Source: Front Public Health. 2025 Oct 8;13:1620477. doi: 10.3389/fpubh.2025.1620477 (PMC12540475; doi:10.3389/fpubh.2025.1620477)
Supplement: Supplementary file 2 [file Data_Sheet_2.docx]

Figures for Impact at work and in society

# Impact at work due to MPH

## Management

Figure S1 Impact of MPH on Management practices at work

## Academic

Figure S2 Impact of the MPH on academic functions at work

## Advocacy

Figure S3 Impact of the MPH on advocacy at work

## Social Responsiveness

# Impact on society

Figure S4 Impact in society due to the MPH
