# Supplementary material for: Perceptions of South Africa’s master of public health graduates on the degree’s contribution to their leadership at work and in society
Source: Front Public Health. 2025 Oct 8;13:1620477. doi: 10.3389/fpubh.2025.1620477 (PMC12540475; doi:10.3389/fpubh.2025.1620477)
Supplement: Supplementary file 3 [file Data_Sheet_3.docx]

**S3.1 Table of impacts at work and in society that are NOT applicable for respondents by prior educational qualification**

|  | **Business % (95%CI)** | **Doctor % (95%CI)** | **Nurse % (95%CI)** | **Patient facing practitioner % (95%CI)** | **Humanities % (95%CI)** | **Other health training % (95%CI)** | **Pure science % (95%CI)** | **Total** |
| --- | --- | --- | --- | --- | --- | --- | --- | --- |
| **Number** | 7 | 43 | 33 | 60 | 27 | 15 | 23 | 221 |
| **Impact on management at work** | | | | | | | | |
| Created evidence for decision making | 0,0 | 2,3 (0,03-15,2) | 3,0 (0,41-19,3) | 5,0 (1,6-14,6) | 3,7 (0,5-23,0) | 0,0 | 8,7 (2,1-29,7) | 3,2 (1,5-6,3) |
| Reported on/made recommendations on population needs | 14,2 (1,6-62,4) | 2,3 (0,3-15,2) | 6,1 (1,5-21,8) | 8,3 (3,418,7) | 7,4 (1,8-25,9) | 0,0 | 17,4 (6,5-39,0) | 6,8 (4,2-10,7) |
| Contributed to policy changes at work | 14,3 (1,6-62,4) | 11,6 (4,8-25,4) | 9,1 (2,9-25,2) | 6,7 (2,5-16,7) | 14,8 (5,5-34,1) | 0,0 | 21,7 (9,1-43,5) | 9,1 (6,1-13,4) |
| Policy changes at higher level | 28,5 (6,3-70,4) | 7,0 (2,2-19,8) | 9,1 (2,9-25,2) | 8,3 (3,5-18,7) | 7,4 (1,8-25,9) | 6,7 (0,9-37,1) | 17,4 (6,9-39,0) | 9,2 (6,1-13,4) |
| Participation in high level committees | 14,3 (1,6-62,4) | 7,0 (2,2-19,8) | 3,0 (0,5-20,7) | 10 (4,5-20,7) | 3,7 (0,5-23,0) | 6,7 (0,9-37,1) | 17,4 (6,4-39,0) | 6,8 (4,2-10,7) |
| **Social responsiveness impact at work** | | | | | | | | |
| Developed Interventions based on consultation & evidence | 14,3 (1,6-62,4) | 2,3 (0,3-15,2) | 0,0 | 6,7 (2,5-16,7) | 7,4 (1,8-26,0) | 20,0 (6,3-48,3) | 8,7 (21,0-29,7) | 5,2 (3,0-0-7,7) |
| Implemented improvement strategies | 14,3 (1,6-62,4) | 9,3 (3,5-22,6) | 0,0 | 8,3 (3,5-22,6) | 7,4 (1,8-26,0) | 13,3 (3,2-42,0) | 13,0 (4,1-34,3) | 7,1 (4,6-11,1) |
| Improving human resources management | 14,3 (1,6-62,4) | 11,6 (4,8-25,4) | 6,1 (14,7-21,8) | 6,7 (2,5-16,7) | 7,4 (1,8-25,9) | 26,7 (9,9-54,5) | 30,4 (14,9-52,2) | 11,2 (7,8-15,7) |
| Overcome inefficiencies | 14,3 (1,6-62,4) | 14,0 (6,3-28,1) | 6,1 (1,5-21,8) | 8,3 (3,5-18,7) | 13,3 (3,1-42,0) | 13,3 (15,0-52,2) | 30,4 (14,9-52,2) | 12,0 (8,5-16,6) |
| Workplace improvements | 14,3 (1,6-62,4) | 9,3 (3,5-22,6) | 3,0 (0,4-19,3) | 8,3 (3,4-18,7) | 11,1 (3,5-30,0) | 13,3 (3,2-42,0) | 34,8 (18,1-56,3) | 10,0 (6,8-14,4) |
| Addressed social determinants of health | 14,3 (1,6-62,4) | 4,7 (1,1-17,1) | 6,1 (1,5-21,8) | 5,0 (1,6-14,6) | 3,7 (0,5-23,0) | 6,7 (0,8-37,1) | 21,7 (9,1-43,5) | 6,4 (3,9-10,2) |
| Worked intersectionally* | 14,3 (1,6-62,4) | 9,3 (3,5-22,6) | 6,1 (1,5-21,8) | 11,7 (5,6-22,7) | 7,4 (1,8-26,0) | 6,7 (0,9-27,1) | 39,1 (21,3-60,3) | 10,8 (7,5-15,3) |
| **Impact on society** | | | | | | | | |
| Changes beyond workplace | 14,3 (1,6-62,4) | 11,6 (4,8-25,4) | 6,1 (1,5-21,8) | 10,0 (4,6-20,7) | 11,1 (3,5-30,0) | 20,0 (6,3-48,3) | 30,4 (14,9-52,2) | 12,0 (8,5-16,6) |
| Change to regulations/law beyond workplace | 42,9 (12,9-79,2) | 11,6 (4,8-25,4) | 6,1 1,5-25,3) | 13,3 (6,7-25,7 | 11,1 (3,5-30,0) | 6,7 (0,9-37,1) | 30,4 (14,9-52,2) | 12,7 (9,1-17,5) |
| Participated in organisations outside of health | 14,3 (1,6-62,4) | 14,0 (6,3-28,1) | 6,1 (1,5-28,0) | 11,7 (5,6-22,7) | 7,4 (1,8-25,9) | 6,7 (0,9-37,1) | 17,4 (6,5-39,0) | 9.9 (6,8-14,4) |
| Contributed to a pro-poor society | 42,9 (12,9-79,2) | 9,3 (3,5-22,6) | 12,1 (4,5-28,6) | 13,3 (6,7-24,7) | 11,1 (3,5-29,9) | 23,3 (3,2-42,0) | 21,7 (9,1-43,5) | 12.0 (0,8-16,6) |
| Contributed to societal equity interventions | 28,5 (6,3-70,4) | 9,3 (3,4-22,6) | 12,1 (4,5 -28,6) | 12,1 (4,5-28,6) | 0,0 | 13,3 (3,2-42,0) | 34,8 (18,1-56,3) | 11,9 (8,4-16,6) |
| Promoted equitable service access | 14,3 (1,6-62,4) | 9,3 (3,5-22,6) | 6,1 (1,5-21,8) | 11,7 (5,6-22,8) | 11,1 (3,5-30,0) | 20,0 (6,3-48,3) | 26,1 (11,9-47,9) | 10,4 (7,1-14,8) |
| Promoted access to quality services | 12,3 (1,6-62,4) | 4,7 (1,1-17,1) | 3,0 (0.4-19,3) | 13,3 (6,7-24,7) | 7,4 (1,8-25,9) | 26,7 (9,9-54,5) | 26,1 (11,9-47,9) | 8,4 (5,5-12,5) |
| Contributed to resource mobilisation for disadvantaged | 14,3 (1,6-62,4) | 11,6 (4,8-25,3) | 6,1 (1,5-21,8) | 10,0 (4,5-20,7) | 14,8 (5,5-34,1) | 26,7 (9,9-54,5) | 30,4 (14,9-52,2) | 12,0 (8,5-16,6) |
| Promote understanding of public health in society | 14,3 (1,6-62,4) | 7,0 (2,2-19,8) | 3,0 (0,4-19,3) | 5,0 (1,6-14,6) | 3,7 (0,5-23,0) | 6,7 (0,9-37,2) | 21,7 (9,1-43,5) | 6,8 (4,2-10,7) |
| * advocacy impact item | lower than overall proportion | |  |  |  |  |  |  |
|  | higher than overall proportion | |  |  |  |  |  |  |

**S3.2 Table of impacts at work and in society that are NOT applicable for respondents by role at work**

|  | **Health Promotion % (95%CI)** | **Academic % (95%CI)** | **Pure research % (95%CI)** | **Line manager % (95%CI)** | **Program manager % (95%CI)** | **Policy % (95%CI)** | **Total** |
| --- | --- | --- | --- | --- | --- | --- | --- |
| **Number** | 21 | 16 | 18 | 23 | 47 | 15 | 221 |
| **Impact on management at work** | | | | | | | |
| Created evidence for decision making | 0,0 | 6,3 (0,8-35,4) | 0,0 | 0,0 | 2,1 (0,3-14,0) | 6,7 (0,9-37,2 | 3,2 (1,5-6,3) |
| Reported on/made recommendations on population needs | 14,3 (4,5-37,0) | 12,5 (3,0-40,0) | 5,6 )0,7-32,2) | 4,3 (0,6-26,6) | 2,2 (0,3-14,0) | 0,0 | 6,8 (4,2-10,7) |
| Contributed to policy changes at work | 14,3 (4,5-37,0) | 18,8 (5,9-46,0) | 22,2 (8,3-47,4) | 0,0 | 6,4 (2,0-18,3) | 6,7 (0,9-37,25) | 9,1 (6,1-13,4) |
| Policy changes at higher level | 14,3 (4,5-37,0) | 31,3 (13,1-57,7) | 5,6 (0,7-32,2) | 4,3 (0,6-26,4) | 2,1 (0,3-14,0) | 6,7 (0,9-37,2) | 9,2 (6,1-13,4) |
| Participation in high level committees | 9,5 (2,3-32,1) | 12,5 (3,0-32,1) | 5,6 (0,7-32,2) | 8,7 (2,1-29,8) | 4,3 (1,0-15,8) | 6,6 (0,9-37,2) | 6,8 (4,2-10,7) |
| **Social responsiveness impact at work** | | | | | | | |
| Developed Interventions based on consultation & evidence | 4,7 (0,6-28,4) | 6,3 (0,8-35,4) | 6,3 (0,8-35,4) | 0,0 | 0,0 | 6,7 (8,5-37,2) | 5,2 (3,0-0-7,7) |
| Implemented improvement strategies | 0,0 | 12,5 (3,0-40,0) | 27,8 (11,7-52,8) | 0,0 | 0,0 | 6,7 (0,8-37,1) | 7,1 (4,6-11,1) |
| Improving human resources management | 23,8 (10,0-46,8) | 18,8 (5,9-46,0) | 33,3 (15,3-58,0) | 0,0 | 2,1 (0,3-14,0) | 6,7 (0,9-37,2) | 11,2 (7,8-15,7) |
| Overcome inefficiencies | 23,8 (10,0-46,8) | 18,8 (5,9-46,0) | 27,8 (11,7-52,8) | 4,3 (0,6-26,4) | 2,1 (0,3-14,0) | 6,7 (0,9-37,2) | 12,0 (8,5-16,6) |
| Workplace improvements | 14,3 (4,5-37,0) | 12,5 (3,0-40,0) | 27,8 (11,7-52,8) | 0,0 | 4,3 (1,0-15,8) | 13,3 (3,2-42,1) | 10,0 (6,8-14,4) |
| Addressed social determinants of health | 4,8 (0,6-28,4) | 0,0 | 22,2 (8,3-47,5) | 0,0 | 2,1 (0,3-14,0) | 6,7 (0,9-37,2) | 6,4 (3,9-10,2) |
| Worked intersectionally* | 9,5 (2,3-2,1) | 12,5 (3,0-40,0) | 27,8 (11,7-52,8) | 4,3 (0,6-37,2) | 6,4 (2,0-18,3) | 6,7 (0,9-37,2) | 10,8 (7,5-15,3) |
| **Impact on society** | | | | | | | |
| Changes beyond workplace | 14,3 (4,5-37,0) | 18,8 (5,9-46,0) | 16,7 (5,2-42,0) | 4,3 (0,6-26,4) | 8,5 (3,2-20,9) | 6,7(0,9-37,2) | 12,0 (8,5-16,6) |
| Change to regulations/law beyond workplace | 14,2 (4,5-37,0) | 18,8 (5,9-46,0) | 16,7 (5,2-41,9) | 8,7 (2,1-18,3) | 6,4 (2,0-18,3) | 6,7 (0,9-37,2) | 12,7 (9,1-17,5) |
| Participated in organisations outside of health | 4,8 6,3-28,4) | 18,8 (5,9-46,0) | 27,8 (11,7-52,8) | 8,7 (2,1-29,8) | 8,5 (3,2-20,9) | 6,7 (0,9-37,2) | 9.9 (6,8-14,4) |
| Contributed to a pro-poor society | 19,1 (7,1-52,0) | 12,5 (3,0-40,0) | 33,3 (15,3-58,0) | 8,7 (2,1-18,3) | 6,4 (2,0-18,3) | 6.3 (0,9-37,2) | 12.0 (0,8-16,6) |
| Contributed to societal equity interventions | 14,2 (4,5-37,0) | 12,5 (3,0-40,0) | 16,7 (5,2-42,0) | 8,7 (2,1-29,8) | 4,3 (1,0-15,8) | 6,7 (0,9-37,2) | 11,9 (8,4-16,6) |
| Promoted equitable service access | 14,3 (4,5-37,0) | 12,5 (3,0-40,0) | 27,8 (11,7-52,8) | 4,3 (0,6-26,4) | 4,2 (1,0-15,8) | 13,3 (3,2-42,1) | 10,4 (7,1-14,8) |
| Promoted access to quality services | 9,5 (2,3-32,1) | 6,3 (0,8-35,4) | 22,2 (8,3-47,5) | 0,0 | 4,3 (1,0-15,8) | 13,3 (3,2-42,1) | 8,4 (5,5-12,5) |
| Contributed to resource mobilisation for disadvantaged | 23,8 (10,0-46,8) | 18,8 (5,9-46,0) | 27,8 (11,7-52,8) | 4,3 (0,6-26,4) | 4,3 (1,0-15,8) | 6,7 (0,9-37,2) | 12,0 (8,5-16,6) |
| Promote understanding of public health in society | 4,8 (0,6-28,4) | 6,3 (0,8-35,4) | 22,2 (8,2-35,4) | 0,0 | 4,3 (1,0-15,8) | 13,3 (3,2-42,1) | 6,8 (4,2-10,7) |
| advocacy impact item | lower than overall proportion | |  |  |  |  |  |
|  | higher than overall proportion | |  |  |  |  |  |

**S3.3 Table of leadership and impact scores by prior profession**

|  | **Business x̅ (s)** | **Doctor x̅ (s)** | **Nurse x̅ (s)** | **Patient facing practitioner x̅ (s)** | **Humanities x̅ (s)** | **Other health training x̅ (s)** | **Pure science x̅ (s** | **Overall x̅ (s)** |
| --- | --- | --- | --- | --- | --- | --- | --- | --- |
| **Leadership** | | | | | | | | |
| Number | 3 | 25 | 14 | 33 | 19 | 8 | 12 | 122 |
| Leadership skills applicability to work (n=123) | 13,33 (2,89) | 11,76 (2,77) | 10,71 (3,73) | 10,67 (3,79) | 12,4 (2,3) | 14 (1,8) | 11,7 (3,1) | 11,75 (3,19) |
| Leadership skills from MPH (n=122) | 11,0 (6,93) | 6,72 (4,73) | 7,29 (5,41) | 6,79 (5,26) | 7,47 (5,35) | 11,25 (4,49) | 7,58 (4,58) | 7,60 (5,13) |
| **Impact on management at work** | | | | | | | | |
| Number | 3 | 28 | 17 | 38 | 19 | 7 | 14 | 134 |
| Created evidence for decision making (n=126) | 1,67 (0,58) | 1,19 (0,74) | 1,31 (0,60) | 1,23 (0,60) | 1,17 (0,71) | 1,86 (0,38) | 1,17 (0,72) | 1,27 (0,67) |
| Reported on/made recommendations on population needs (n=116) | 1,67 (0,58) | 1,19 (0,74) | 1,31 (0,60) | 1,23 (0,60) | 1,17 (0,71) | 1,86 (0,38) | 1,17 (0,72) | 1,41 (0,63) |
| Contributed to policy changes at work (n=111) | 2,00 (0,00) | 1,14 (0,77) | 1,50 (0,65) | 1,32 (0,68) | 1,06 (0,77) | 1,71 (0,76) | 1,22 (0,67) | 1,29 (0,73) |
| Policy changes at higher level (n=111) | 1,00 (-) | 1,17 (0,82) | 1,50 (0,65) | 1,18 (0,64) | 1,06 (0,64) | 1,83 (0,41) | 1,1 (0,57) | 1,23 (0,69) |
| Participation in high level committees (n=117) | 1,00 (-) | 1,25 (0,79) | 1,63 (0,50) | 1,47 (0,62) | 1,42 (0,69) | 2,00 (-) | 1,40 (0,70) | 1,47 (0,65) |
| **Social responsiveness impact at work** | | | | | | | | |
| Number | 3 | 26 | 17 | 38 | 17 | 7 | 14 | 130 |
| Developed Interventions based on consultation & evidence (n=117) | 2,00 (0,00) | 1,16 (0,69) | 1,47 (0,62) | 1,09 (0,57) | 1,20 (0,68) | 1,25 (0,96) | 1,33 (0,65) | 1,05 (0,92) |
| Implemented improvement strategies (n=111) | 2,00 (0,00) | 1,18 (0,73) | 1,35 (0,70) | 1,06 (0,70) | 1,07 (0,88) | 1,60 (0,55) | 1,50 (0,53) | 1,25 (0,72) |
| Improving human resources management (n=102) | 2,00 (0,00) | 1,24 (0,70) | 1,27 (0,70) | 1,09 (0,71) | 0,80 (0,77) | 1,00 (0,00) | 1,14 (0,69) | 1,15 (0,72) |
| Overcome inefficiencies (n=98) | 2,00 (0,00) | 1,20 (0,89) | 1,27 (0,70) | 1,19 (0,65) | 0,92 (0,76) | 0,80 (0,83) | 0,86 (0,69) | 1,16 (0,76) |
| Workplace improvements (n=105) | 2,00 (0,00) | 1,23 (0,81) | 1,43 (0,73) | 1,30 (0,690 | 1,21 (0,70) | 1,40 (0,89) | 0,86 (0,75) | 1,26 (0,75) |
| Addressed social determinants of health (n=114) | 2,00 (0,00) | 1,25 (0,74) | 1,33 (0,62) | 1,20 (0,72) | 1,50 (0,63) | 1.67 (0,82) | 1,56 (0,53) | 1,38 (0,70) |
| Worked intersectionally* (n=105) | 2,00 (-) | 1,09 (0,81) | 1,33 (0,49) | 1,23 (0,67) | 1,06 (0,68) | 1,71 (0,76) | 1,60 (0,89) | 1,27 (0,71) |
| **Impact on society** | | | | | | | | |
| Number | 3 | 26 | 17 | 38 | 17 | 7 | 14 | 130 |
| Changes beyond workplace (n=99) | 1,00 (0,00) | 1,10 (0,83) | 1,20 (0,68) | 1,13 (0,61) | 0,71 (0,72) | 1,75 (0,50) | 1,67 (0,52) | 1,12 (0,72) |
| Change to regulations/law beyond workplace (n=97) | 0 | 1,05 (0,80) | 1,21 (0,80) | 1,10 (0,71) | 0,93 (0,73) | 1,67 (0,52) | 1,29 (0,95) | 1,13 (0,76) |
| Participated in organisations outside of health (n=105) | 1,00 (0,00) | 1,05 (0,76) | 1,33 (0,90) | 1,16 (0,69) | 1,00 (0,76) | 1,33 (0,82) | 0,80 (0,79) | 1,13 (0,76) |
| Contributed to a pro-poor society (n=100) | 0 | 1,05 (0,79) | 1,38 (0,65) | 1,03 (0,72) | 0,93 (0,62) | 1,20 (0,84) | 0,56 (0,73) | 1,03 (0,74) |
| Contributed to societal equity interventions (n=98) | 1,00 (-) | 1,14 (0,77) | 1,46 (0,52) | 1,17 (0,65) | 0,87 (0,52) | 1,40 (0,89) | 1,17 (0,41) | 1,16 (0,67) |
| Promoted equitable service access (n=104) | 2,00 (0) | 1,05 (0,79) | 1,33 (0,49) | 1,19 (0,65) | 1,00 (0,55) | 1,50 (0,58) | 0,63 (0,74) | 1,16 (0,70) |
| Promoted access to quality services(n=107) | 2,00 (0,00) | 1,21 (0,78) | 1,19 (0,54) | 1,18 (0,73) | 1,00 (0,41) | 1,67 (0,58) | 0,50 (0,76) | 1,18 (0,71) |
| Contributed to resource mobilisation for disadvantaged (n=100) | 2,00 (0,00) | 1,24 (0,77) | 1,20 (0,68) | 1,19 (0,74) | 1,16 (0,55) | 1,67 (0,58) | 0,71 (0,76) | 1,23 (0,72) |
| Promote understanding of public health in society (n=112) | 2,00 (0,00) | 1,39 (0,66) | 1,38 (0,50) | 1,09 (0,75) | 1,31 (0,70) | 1,29 (0,76) | 0,75 (0,71) | 1,28 (0,70) |
| * advocacy impact item |  | Significantly lower mean score | | |  | Significantly higher mean score | | |

**S3.4 Table of leadership and impact scores by roles at work**

|  | **Program manager x̅ (s)** | **Health service manager x̅ (s)** | **Pure research x̅ (s)** | **Health Promotion/ communicat x̅ (s)** | **Academic x̅ (s)** | **Policy x̅ (s)** | **Overall x̅ (s)** |
| --- | --- | --- | --- | --- | --- | --- | --- |
| **Leadership** | | | | | | | |
| Number | 25 | 10 | 10 | 16 | 9 | 11 | 122 |
| Leadership skills applicability to work (n=123) | 11,92 (3,12) | 10,9 (3,00) | 8,0 (4,11) | 11,67 (1,87) | 11,67 (1,87) | 13,2 (2,2) | 11,75 (3,19) |
| Leadership skills from MPH (n=122) | 7,16 (4,84) | 5 ,00(4,54) | 3,80 (3,36) | 9 ,00 (5,45) | 7,22 (5,23) | 9,10 (5,19) | 7,60 (5,13) |
| **Impact on management at work** | | | | | | | |
| Number | 27 | 12 | 13 | 17 | 10 | 12 | 134 |
| Created evidence for decision making (n=126) | 1,31 (0,62) | 1,25 (0,45) | 1,31 (0,48) | 1,35 (0,70) | 0,89 (0,60) | 1,18 (0,75) | 1,27 (0,67) |
| Reported on/made recommendations on population needs (n=116) | 1,22 (0,64) | 1,81 (0,40) | 1,42 (0,67) | 1,77 (0,44) | 1,38 (0,74) | 1,50 (0,67) | 1,41 (0,63) |
| Contributed to policy changes at work (n=111) | 1,32 (0,69) | 1,25 (0,45) | 1,00 (1,00) | 1,36 (0,74) | 1,00 (0,82) | 1,45 (0,52) | 1,29 (0,73) |
| Policy changes at higher level (n=111) | 1,33 (0,68) | 1,36 (0,50) | 1,17 (0,58) | 1,00 (0,68) | 1,20 (0,84) | 1,45 (0,69) | 1,23 (0,69) |
| Participation in high level committees (n=117) | 1,46 (0,65) | 1,40 (0,52) | 1,33 (0,65) | 1,8 (0,41) | 1,00 (0,76) | 1,73 (0,47) | 1,47 (0,65) |
| **Social responsiveness impact at work** | | | | | | | |
| Number | 27 | 12 | 12 | 16 | 10 | 12 | 130 |
| Developed Interventions based on consultation & evidence (n=117) | 1,19 (0,62) | 1,25 (0,62) | 0,88 (0,35) | 1,67 (0,49) | 1,11 (0,93) | 1,45 (0,69) | 1,05 (0,92) |
| Implemented improvement strategies (n=111) | 1,32 (0,63) | 1,00 (0,60) | 0,86 (0,69) | 1,56 (0,63) | 1,00 (0,76) | 1,36 (0,67) | 1,25 (0,72) |
| Improving human resources management (n=102) | 1,92 (0,69) | 0,92 (0,51) | 0,83 (0,41) | 1,54 (0,82) | 1,57 (0,53) | 1,45 (0,69) | 1,15 (0,72) |
| Overcome inefficiencies (n=98) | 1,33 (0,76) | 1,18 (0,60) | 0,86 (0,38) | 1,45 (0,82) | 0,86 (0,90) | 1,27 (0,79) | 1,16 (0,76) |
| Workplace improvements (n=105) | 1,28 (0,79) | 1,25 (0,62) | 1,29 (0,49) | 1,54 (0,78) | 1,25 (0,89) | 1,20 (0,79) | 1,26 (0,75) |
| Addressed social determinants of health (n=114) | 1,38 (0,64) | 1,25 (0,62) | 1,38 (0,75) | 1,60 (0,51) | 1,50 (0,85) | 1,45 (0,69) | 1,38 (0,70) |
| Worked intersectionally* (n=105) | 1,20 (0,76) | 0,91 (0,30) | 0,86 (0,38) | 1,43 (0,64) | 1,38 (0,92) | 1,73 (0,47) | 1,27 (0,71) |
| **Impact on society** | | | | | | | |
| Number | 27 | 12 | 12 | 16 | 10 | 12 | 130 |
| Changes beyond workplace (n=99) | 1,18 (0,59) | 1,09 (0,54) | 0,89 (0,78) | 1,08 (0,76) | 1,14 (0,69) | 1,45 (0,82) | 1,12 (0,72) |
| Change to regulations/law beyond workplace (n=97) | 1,00 (0,66) | 1,00 (0,71) | 1,22 (0,83) | 1,23 (0,83) | 1,14 (0,69) | 1,55 (0,69) | 1,13 (0,76) |
| Participated in organisations outside of health (n=105) | 1,09 (0,73) | 1,10 (0,74) | 1,29 (0,95) | 1,40 (0,74) | 1,29 (0,95) | 1,27 (0,65) | 1,13 (0,76) |
| Contributed to a pro-poor society (n=100) | 1,00 (0,72) | 0,90 (0,74) | 1,33 (0,52) | 1,58 (0,67) | 1,00 (0,93) | 1,18 (0,75) | 1,03 (0,74) |
| Contributed to societal equity interventions (n=98) | 1,24 (0,60) | 1,20 (0,63) | 1,00 (0,71) | 1,42 (0,67) | 1,13 (0,83) | 1,20 (0,63) | 1,16 (0,67) |
| Promoted equitable service access (n=104) | 1,28 (0,61) | 1,81 (0,60) | 1,43 (0,53) | 1,38 (0,77) | 0,88 (0,83) | 1,10 (0,74) | 1,16 (0,70) |
| Promoted access to quality services(n=107) | 1,40 (0,71) | 1,08 (0,51) | 1,13 (0,35) | 1,62 (0,51) | 0,78 (0,83) | 1,20 (0,79) | 1,18 (0,71) |
| Contributed to resource mobilisation for disadvantaged (n=100) | 1,44 (0,71) | 1,00 (0,63) | 1,14 (0,38) | 1,55 (0,69) | 1,29 (0,95) | 1,18 (0,75) | 1,23 (0,72) |
| Promote understanding of public health in society (n=112) | 1,42 (0,72) | 1,17 (0,58) | 1,38 (0,52) | 1,33 (0,82) | 1,67 (0,50) | 1,30 (0,48) | 1,28 (0,70) |
|  |  | Significantly lower mean score | | |  | Significantly higher mean score | |
